# The effects of on-pump and off-pump coronary artery bypass surgery on oxidative stress and cerebral oxygenation: a prospective observational study

**DOI:** 10.55730/1300-0144.5770

**Published:** 2023-12-14

**Authors:** Ökkeş Hakan MİNİKSAR, Sameh ALAGHA, Ferit ÇİÇEKÇİOĞLU, Mehtap HONCA, Ayse Yeşim GÖÇMEN

**Affiliations:** 1 Department of Anesthesiology and Reanimation, Dr Abdurrahman Yurtaslan Oncology Training and Education Hospital, University of Health Sciences, Ankara, Turkiye; 2 Department of Cardiovascular Surgery, Faculty of Medicine, Yozgat Bozok University, Yozgat, Turkiye; 3 Department of Cardiovascular Surgery, Etlik City Hospital, University of Health Sciences, Ankara, Turkiye; 4 Department of Anesthesiology and Reanimation, Bilkent City Hospital, University of Health Sciences, Ankara, Turkiye; 5 Department of Biochemistry, Faculty of Medicine, Yozgat Bozok University, Yozgat, Turkiye

**Keywords:** Cardiopulmonary bypass, off-pump coronary artery bypass grafting, oxidative stress, cerebral oxygenation

## Abstract

**Background/aim:**

In this prospective observational study, our goal was to investigate the relationship between serum levels of oxidative stress (OS) parameters and regional cerebral oxygen saturation (rSO_2_) in addition to evaluating postoperative clinical outcomes among patients undergoing coronary artery bypass graft surgery (CABG).

**Materials and methods:**

This study comprised 64 adult patients undergoing elective CABG (on-pump [n = 48] and off-pump [n = 16]) procedures. Serum OS levels and rSO_2_ values were measured intraoperatively at three specific time points: T1 (after induction), T2 (15 min before aortic cross-clamp removal or the final distal anastomosis), and T3 (15 min after aortic cross-clamp removal or the last distal anastomosis).

**Results:**

Serum OS and lactate values demonstrated higher levels at T2 and T3 (p < 0.001), while rSO_2_ values were lower at T2 (p = 0.024) in the on-pump CABG group compared to the off-pump CABG group. The rSO_2_ values at T2 exhibited a negative correlation with OS parameters, lactate levels at T2 and T3, aortic clamp time, postoperative mechanical ventilation time, and intensive care unit stay length. In the multivariate linear regression analysis (R2 = 0.181, p = 0.001), lactate values at T2 emerged as the sole factor affecting the OS index at T2 (t = 2.843, p = 0.006).

**Conclusion:**

In our study, we observed elevated OS values and relatively low rSO_2_ values during on-pump CABG procedures, with rSO_2_ showing an association with increased OS parameters. Close monitoring of the OS response level and rSO_2_ during CABG could potentially enhance postoperative clinical outcomes.

## 1. Introduction

On-pump coronary artery bypass graft surgery (on-pump CABG), conducted on the arrested heart using a cardiopulmonary bypass (CPB) machine, presents several advantages, particularly for the surgeon. It provides a secure and comfortable operating field and can alleviate various forms of organ damage, especially myocardial ischemia [1]. However, the CPB machine has certain disadvantages, including nonpulsatile flow, contact of circulating blood with silicone tube systems, and the potential for blood cell fragmentation throughout the roller pump system [2]. In contrast, off-pump CABG is performed on the beating heart without the use of CPB, thereby avoiding extracorporeal blood circulation [1,2]. Consequently, off-pump CABG has been associated with a shorter duration of mechanical ventilation (MV), a lower incidence of end-organ damage, and shorter lengths of stay in the intensive care unit (ICU) and hospital. [1].

Oxidative stress (OS) is defined as “the deterioration of oxidative balance caused by the increase in reactive oxygen species (ROS) formed during cellular metabolism and the insufficient presence of antioxidants” [3]. Oxidant-antioxidant capacity can be measured in vivo using plasma biomarkers such as malondialdehyde (MDA), total oxidant stress (TOS), superoxide dismutase (SOD), total antioxidant stress (TAS), and glutathione peroxidase (GPx) [2–5]. Myocardial ischemia and reperfusion during cardiac surgery serve as potent sources of ROS, including superoxide radicals, hydroxyl radicals, and hydrogen peroxide (H2O2), which are associated with adverse clinical outcomes [3,4]. However, several studies have reported that on-pump CABG induces a more pronounced OS response compared to off-pump CABG, and this heightened response is associated with various surgical complications [1,2,5].

Near-infrared spectroscopy (NIRS) provides continuous and noninvasive measurements of regional cerebral hemoglobin oxygen saturation (rSO_2_) in brain tissue, particularly during general anesthesia [6]. The widespread use of intraoperative NIRS aims to detect adverse clinical outcomes earlier, particularly in major surgeries such as cardiac surgery [7]. In our recent observational study, we discovered that low rSO_2_ serves as a risk factor for perioperative neurocognitive disorder in cardiac surgery patients [8]. Other studies have identified significant declines in rSO_2_ as being associated with increased major organ dysfunction and prolonged lengths of stay in the ICU and hospital [9]. However, it has been reported that cerebral desaturation does not differ at any time between patients with and without the use of CPB [10].

Hypoxic conditions stimulate an overproduction of ROS, leading to an OS response. Consequently, the excessive ROS production triggers the oxidation of biological molecules, including DNA, lipids, and proteins [3,11]. Furthermore, cerebral hypoperfusion, which induces OS and inflammation in the brain, has previously been associated with cognitive impairment and neuronal cell damage [3,4,8–11]. On-pump CABG is associated with a heightened OS response [1,2,5] and lower rSO_2_ [6,7,10] compared to off-pump CABG. Additionally, the excessive systemic inflammatory response during CPB also induces oxidative stress [4,12]. Therefore, we hypothesized that intraoperative OS arising from impaired cerebral autoregulation, cerebral hypoperfusion, myocardial ischemia-reperfusion, and hypoxia in cardiac surgery patients, varies with the type of cardiac surgery and may be associated with rSO_2_ values. Understanding the mechanisms behind these effects may aid in preventing adverse outcomes in cerebral oxygenation for patients undergoing high-risk cardiac bypass surgery. To our knowledge, no published clinical trials have evaluated the relationship between OS and rSO_2_ levels based on the type of cardiac surgery.

The primary objective of this study was to compare serum OS parameters and rSO_2_ values between on-pump and off-pump elective CABG procedures at various intraoperative time points. The secondary goals included examining the relationship between serum OS parameters and rSO_2_, as well as assessing the impact on clinical outcomes during CPB and identifying factors affecting intraoperative OS. The results of this study may provide valuable insights for clinicians in making informed decisions regarding the selection of the appropriate surgical procedure in clinical practice.

## 2. Materials and methods

### 2.1. Study design

This prospective, single-center, observational study was conducted at the Medical Faculty Hospital between January 15 and September 30, 2021. Approval for the study was obtained from the Ethics Committee of Yozgat Bozok University (protocol number: KAEK-89_2021.01.18_15; chairperson: Dr. A. C. Aydın) on January 15, 2021. The study adhered to the guidelines outlined in the Helsinki Declaration throughout its duration. Additionally, it was registered on ClinicalTrials.gov with the ID code NCT04834219. The reporting of this prospective observational study follows the Strengthening the Reporting of Observational Studies in Epidemiology (STROBE) guidelines for observational studies [13].

### 2.2. Study population

This study enrolled 64 adult patients aged 30–80 years who provided written informed consent, underwent elective CABG between the specified dates, and met the inclusion criteria. The patients were categorized into two groups based on the use of CPB: the on-pump CABG group (CABG with CPB; n = 48) and the off-pump CABG group (CABG on the beating heart without CPB; n = 16). The groups were compared, with a specific focus on the OS parameter levels and rSO_2_ values at different intraoperative time points.

**Figure 1:**
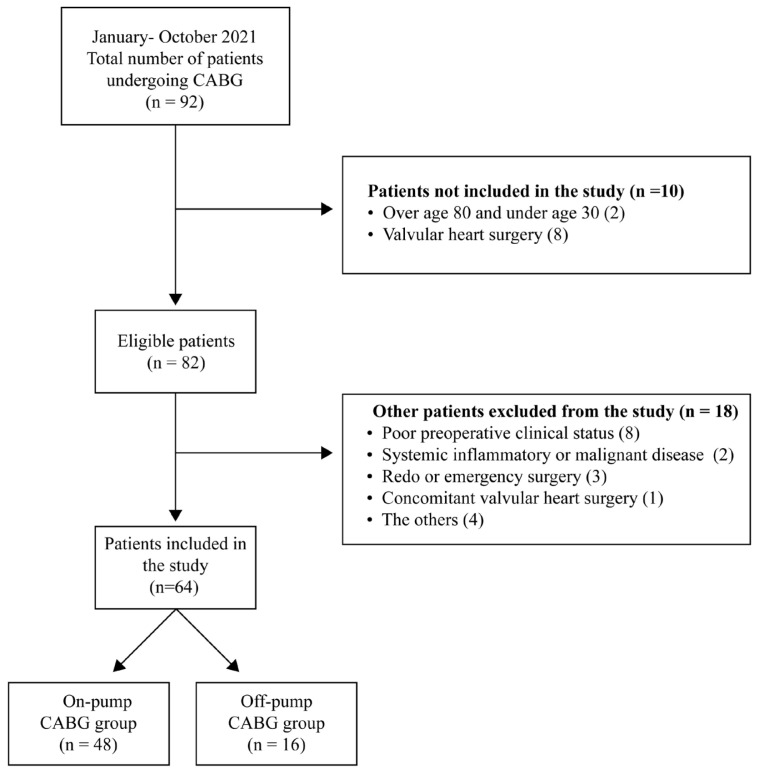
Flow diagram of the study.

This study’s inclusion criteria were: (1) individuals aged 30–80 years and (2) those scheduled for either on-pump or off-pump CABG. Exclusion criteria included: (1) poor preoperative clinical status (such as chronic kidney failure, liver failure, chronic obstructive pulmonary disease, metabolic diseases other than diabetes mellitus, cerebrovascular disease, or active infection), (2) systemic inflammatory or malignant disease, (3) redo or emergency surgery, (4) concurrent valvular heart surgery, (5) unilateral carotid artery stenosis >70% and bilateral carotid artery stenosis >50%, (6) conversion from off-pump to on-pump CABG during the operation, and (7) use of medications, vitamins, immunosuppressive drugs, or dietary supplements with potential antioxidant activity (Figure 1).

### 2.3. Clinical data

The patients’ demographic data, including age, sex, smoking status, and comorbidities, was documented before the surgery. Preoperative transthoracic echocardiography was conducted, and the left ventricular ejection fraction was calculated.

All patients included in the study underwent CABG surgery at our center, performed by the same surgical team with extensive experience in both off-pump and on-pump CABG surgery. The choice between on-pump or off-pump CABG was influenced by individual patient demographics and clinical profiles, taking into consideration factors such as age, diabetes mellitus status, renal function, left ventricular ejection fraction, aortic and coronary vasculatures, presence of chronic obstructive pulmonary disease, and estimated surgical risks. However, the final decision regarding the surgical technique was ultimately determined after evaluating the coronary anatomy and ascending aorta during the surgery.

**Figure 2:**
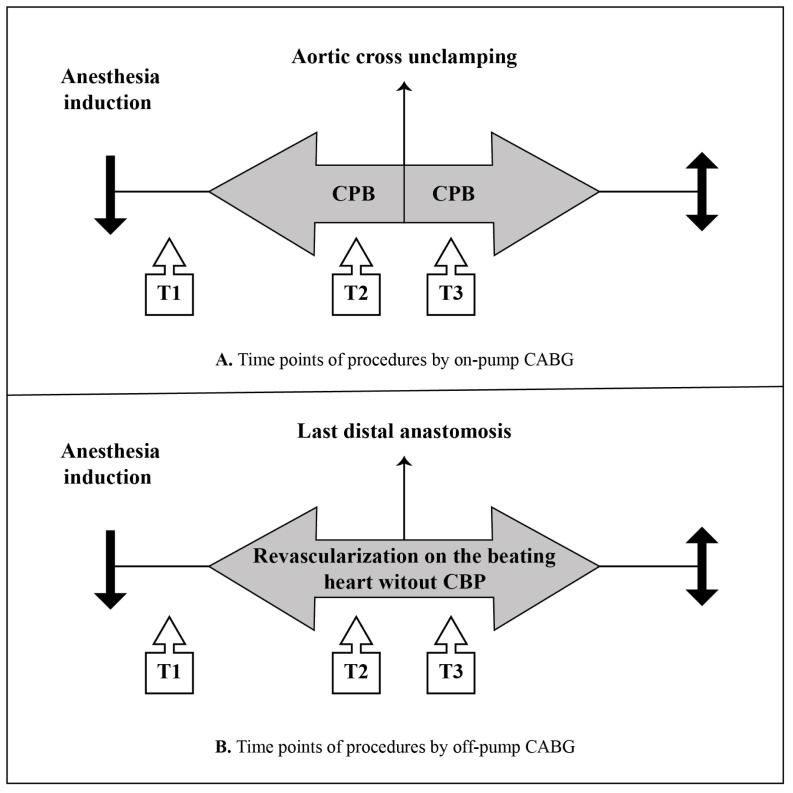
Time points of procedures by on-pump and off-pump CABG groups. T1: after induction and before skin incision (baseline), T2: 15 min before aortic cross-clamp removal (on-pump CABG), 15 min before the last distal anastomosis (off-pump CABG), T3: 15 min after aortic cross-clamp removal (on-pump CABG), 15 min after the last distal anastomosis (off-pump CABG). CPB: cardiopulmonary bypass.

Data were collected and analyzed at three time points: T1, after induction and before skin incision (baseline); T2, 15 min before aortic cross-clamp removal (on-pump CABG) or 15 min before the last distal anastomosis (off-pump CABG); T3, 15 min after aortic cross-clamp removal (on-pump CABG) or 15 min after the last distal anastomosis (off-pump CABG; Figure 2). At all these time points, the patients’ serum OS biomarker levels (MDA, TOS, SOD), rSO_2_, partial pressure of oxygen (PaO_2_), arterial oxygen saturation (SaO_2_), hematocrit (Hct) level, blood glucose level, and lactate level were recorded. Additionally, aortic cross-clamp and CPB time, total operative procedure duration (defined as the time between skin incision and closure), amount of blood transfusion administered, postoperative MV time, and length of stay in the ICU were recorded.

### 2.4. Surgical methods

#### 2.4.1. Anesthetic management

No premedication for sedation purposes was administered to the patients. Upon arrival in the operating room, standard monitoring (electrocardiography, pulse oximetry, and non-invasive blood pressure) was performed, and two peripheral intravenous (IV) cannulas (18 and 16 gauge) were inserted into the upper extremities. The left radial artery cannula was inserted and monitored. Following preoxygenation with 100% O_2_ for 3 min, anesthesia was induced with 2 mg/kg of propofol, 0.3 mg/kg of rocuronium, and 1 μg/kg of fentanyl. The right internal jugular vein was catheterized, and a probe was placed in the nasopharynx to monitor body temperature. In controlled ventilation mode, patients were set to maintain a tidal volume of 8 mL/kg, a fraction of inspired oxygen of 0.6, an inspiratory to expiratory ratio of 1:2, a respiratory rate of 12/min, and an end-tidal carbon dioxide level of 30–40 mmHg. Anesthesia was sustained by inhaling a mixture of 50% O_2_ and 50% air, along with sevoflurane (minimum alveolar concentration of 0.5%–1%). Immediately after anesthesia induction, rSO_2_ probes were placed in the middle of the patient’s forehead, and oxygenation was continuously monitored using an O3 Regional Oximetry device (Masimo, Irvine, CA, USA). A bolus containing 0.05 mg/kg of midazolam and 1 mcg/kg of fentanyl was intermittently administered during CPB to maintain anesthesia depth. All clinical procedures, including the administration of anesthetic drugs, hypothermia induction, blood transfusion, and organ preservation applications, were coordinated by the same team (surgeon, anesthesiologist, and perfusionist) in accordance with standard institutional protocols.

#### 2.4.2. CPB management

All patients underwent CABG surgery under general anesthesia.

##### 2.4.2.1. On-pump CABG

In all patients, a roller pump and membrane oxygenator were used for CPB with biocompatible circuits (Terumo Hardshell Venous Reservoir and Terumo Advanced Perfusion System 1; Ann Arbor, MI, USA). A prime solution, consisting of Isolyte-S (1500 mL), 20% mannitol (100 mL), sodium bicarbonate (10 mEq), and heparin (5000 IU), was prepared. Standard aortic and two-stage venous cannulation was performed. Anticoagulation was achieved with 300 IU/g of heparin, and the active clotting time (ACT) was monitored hourly. CPB was initiated when the ACT exceeded 450 s. CPB was performed at a temperature range of 28–32 °C with moderate hypothermia. Following aortic clamping, cardiac arrest was induced using an antegrade and retrograde cold blood cardioplegia solution (Isolyte-S [500 ccs], potassium chloride [40 mEq], magnesium [12 mEq], and patient blood [500cc]). The hematocrit level was maintained between 20% and 25% during CPB. Before removing the cross-clamp, a final cardioplegia “hotshot” (patient blood [500–700 ccs]) was administered at 37 °C and a dose of 10 mL/kg to reduce the risk of reperfusion injury and provide controlled perfusion. Proximal anastomoses were performed with the assistance of partial aortic occlusion by placing a side clamp during the warming-up phase. Protamine was slowly administered to neutralize heparin at a ratio of 1:1–1.3 mg/mg relative to the heparin dose. Additional protamine was administered as needed to return the ACT to baseline.

##### 2.4.2.2. Off-pump CABG

Following median sternotomy and graft harvesting using the standard technique, intravenous heparin at 1 mg/kg was administered after internal mammary artery mobilization to maintain an ACT of >250 s during anastomosis. A tissue stabilizer (Octopus Tissue Stabilization System; Medtronic, Minneapolis, MN, USA) was used to immobilize the target site of coronary anastomosis. The coronary anastomosis was sequentially performed on the left anterior descending artery, obtuse marginal branch, and posterior descending artery/right coronary artery. During displacement of the beating heart, patients were placed in a 20°–30° head-down position. Norepinephrine was intermittently infused at 0.03–0.05 mg/kg/min if mean arterial pressure (MAP) decreased below 60 mmHg, and epinephrine at 0.03–0.05 mg/kg/min was used intermittently in case of bradycardia. Intracoronary shunts were used to maintain the patency of the coronary arteries during anastomosis in all patients. After distal anastomoses were completed, proximal anastomoses were performed using a partial aortic occlusion clamp. Heparin was not neutralized in most patients.

### 2.5. Sample collection and measurement of serum OS biomarker levels

Venous blood samples were collected from each patient at three time points (T1–T3) during the study period. The collected blood samples were centrifuged at 3000 rpm for 5 min and then separated and stored frozen at −80.0 °C until all patient samples were collected. The determination of serum oxidant profiles involved measuring TOS and MDA levels, while antioxidant profiles were determined by measuring TAS, SOD, and GPx levels.

#### 2.5.1. Determination of total TAS and TOS

Serum TAS and TOS levels were determined using the commercial Rel Assay Diagnostics Kits (Mega Tıp, Gaziantep, Turkey), developed by Erel [14]. TAS was measured in serum by generating 2,2′-azino-di-(3-ethylbenzothiazoline sulfonate) radical cations using the commercial TAS Kit, following the manufacturer’s protocol. TOS was measured in accordance with the same protocol. In this method, the oxidants in the sample oxidized the ferrous ion-o-dianisidine complex to ferric ions. Ferric ions form a colorful complex with xylenol orange in an acidic media. The total number of oxidant molecules in the sample correlates with the color intensity, which can be determined spectrophotometrically. The results are expressed as mol H2O2 equivalent/L of serum after calibrating the assay using H_2_O_2_. The MDA level was measured using a colorimetric kit (Cayman Chemical, Ann Arbor, MI, USA).

#### 2.5.2. Determination of SOD and GPx activity

Total SOD activity was measured using the Rel Assay Diagnostics SOD Activity Assay Kit (Mega Tıp) following the manufacturer’s instructions. GPx activities were determined using the modified method of Paglia and Valentine [17]. In this method, GPx activity was coupled to the oxidation of reduced nicotinamide adenine dinucleotide phosphate (NADPH) by glutathione reductase. NADPH oxidation was recorded spectrophotometrically at 340 nm and 37 °C. The absorbance at 340 nm was recorded, and the results are expressed as U/mL [12].

#### 2.5.3. Calculation of the OS index (OSI)

The OSI was calculated using the TOS to TAS ratio as follows: OSI (arbitrary units) = TOS (μmol H_2_O_2_/L)/TAS (mmol Trolox equiv/L) [14].

### 2.6. The postoperative care process

After CABG surgery, all patients were transferred to the cardiovascular surgery ICU while still intubated. Ventilation settings were configured with a tidal volume of 8 mL/kg and a respiratory rate of 12–15 breaths/min. Following routine treatment and care, patients were weaned from the mechanical ventilator when deemed eligible for extubation.

### 2.7. Statistical analysis

The data were analyzed using SPSS Statistics software (version 25; IBM Corp., Armonk, NY, USA). The normality of each variable’s distribution was assessed using the Kolmogorov–Smirnov and Shapiro–Wilk tests and histograms. Descriptive statistics are presented as mean ± standard deviation (SD) or frequency (percentage). The significance of differences between group means was assessed using chi-square tests, independent two-sample t-tests, paired-sample t-tests, and repeated-measure multivariate analyses. Correlations between variables were assessed using Pearson’s correlation coefficient and linear regression (LR) analyses. The effects of categorical variables (dummy variables: surgery type [on-pump vs. off-pump CABG]) and covariates (rSO_2_ and lactate level at T2 [lactate-T2]) as independent variables on OSIs (at T2 and T3) were examined using the backward LR model (adjusted R^2^), while adjusting for potential confounding factors. The surgery type (on-pump vs. off-pump CABG), which is a categorical variable, was converted into a dummy variable and included in the regression analysis. Model fit in the regression analysis was assessed using appropriate residual and goodness-of-fit statistics. Correlations between intraoperative rSO_2_, OS biomarkers, and other variables were examined using Pearson’s correlation coefficient in each CABG group. All comparative analyses were two-tailed, and a p-value of <0.05 was considered statistically significant.

### 2.8. Sample size

The sample size was estimated using G*Power software (version 3.1.9; Kiel, Germany). For the sample size calculation, we considered OS parameters and rSO_2_ values between on-pump and off-pump CABG surgeries for primary outcomes, based on findings from previous studies [2,10,15]. Considering an error margin of 20%, a confidence interval of 95%, a power of 85%, an effect size of 0.8, and a two-sided significance level, we estimated that a minimum sample size of 56 patients was needed.

## 3. Results

### 3.1. Study population and outcomes

This study comprised 64 patients, with 48 (75%) undergoing on-pump CABG (39 males) and 16 undergoing off-pump CABG (11 males). The mean age was 60.41 ± 10.05 years. Of the participants, 50 (78.1%) were male, and 39 (60.9%) were smokers. Table 1 displays the demographic and perioperative characteristics of patients in the on-pump and off-pump CABG groups. There were significant differences between the on-pump and off-pump groups in terms of blood transfusion amount and postoperative mechanical ventilation duration (Mean = 1.77 units ± 2.1 vs 0.88 units ± 1.1, p = 0.009 and 3.42 hours ± 1 vs 2.5 hours ± 11.3), respectively, (Table 1).

### 3.2. Intraoperative OS parameters

**Figure 3:**
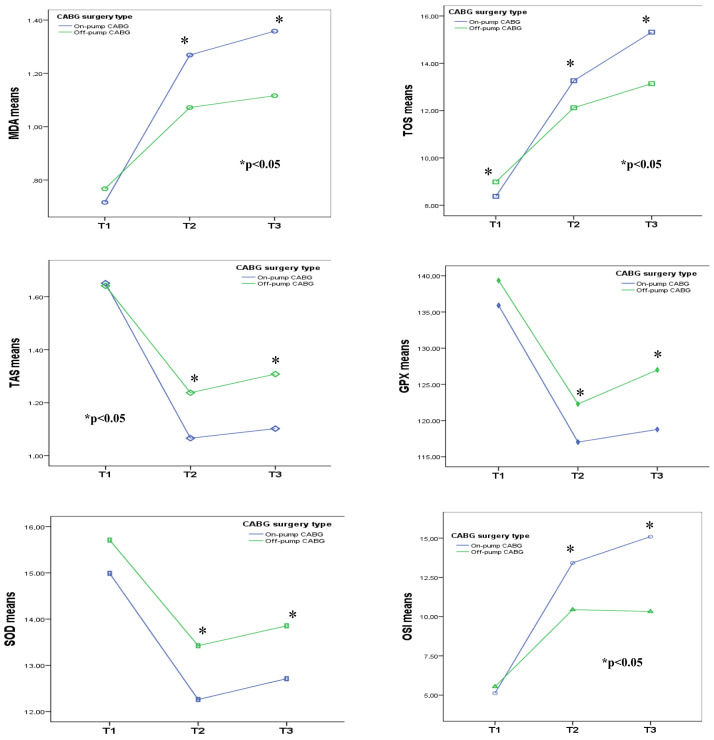
Mean MDA, TAS, TOS, OSI index, SOD, GPX values at different time points (T1, T2, T3) according to the CABG group. MDA, malondialdehyde; SOD, superoxide dismutase; GPX, glutathione peroxidase; TAS, total antioxidant status; TOS, total oxidant status; OSI, oxidative stress index. ^#^p < 0.05; Repeated multivariate tests by different time points. *p < 0.05; Paired tests for on-pump vs. off-pump CABG groups at the same time point.

While the mean levels of MDA, TOS, TAS, and OSI significantly differed between groups at time points T2 and T3, the mean activities of GPx and SOD showed nonsignificant differences (Table 2, Figure 3). MDA-T2-3, TOS-T1-2-3, TAS-T2-3, OSI-T2-3, GPx-T1-2, and SOD-T2-3 values exhibited significant differences between the on-pump and off-pump CABG groups. The TOS-T2-3 values were higher (p = 0.021 and p < 0.001, respectively), the TAS-T2-3 values were lower (p = 0.013 and p = 0.001, respectively), and the OSI-T2-T3 values were higher (p = 0.035 and p = 0.007, respectively) in the on-pump CABG group compared to the off-pump CABG group (Table 2).

### 3.3. Intraoperative variables

While the mean levels of lactate, Hct, and blood glucose significantly differed between groups at time points T1, T2, and T3, the mean values of rSO_2_, SaO_2_, and PaO_2_ did not show significant differences (Table 3). However, _rSO2-_T2 (p = 0.024), SaO_2_-T3 (p = 0.048), PaO_2_-T3 (p = 0.009), and Hct-T2 (p < 0.001) were lower in the on-pump CABG group compared to the off-pump CABG group. Meanwhile, lactate-T2-3 (p < 0.001) and blood glucose-T2 (p < 0.001) were higher in the on-pump CABG group than in the off-pump CABG group.

### 3.4. The relationship between rSO_2_ and OS

#### 3.4.1. On-pump CABG

TOS-T2: There is a significant negative correlation between TOS-T2 and rSO_2_-T2 (r = −0.312, p < 0.05) (Table 4). No significant correlation is observed with rSO_2_-T3 (r = −0.049, p > 0.05). TOS-T3: TOS-T3 shows a significant negative correlation with both rSO_2_-T2 (r = −0.354, p < 0.05) and rSO2-T3 (r = −0.008, p > 0.05). Additionally, a significant positive correlation is found with TAS-T2 (r = 0.323, p < 0.05). TAS-T2: There is no significant correlation between TAS-T2 and rSO2 values. However, a positive correlation is observed with TAS-T3 (r = 0.216, p > 0.05), while a significant negative correlation exists with TOS-T2 (r = −0.557, p < 0.01) and TOS-T3 (r = −0.411, p < 0.01). TAS-T3: TAS-T3 exhibits a significant positive correlation with TAS-T2 (r = 0.405, p < 0.01) and a significant negative correlation with both rSO_2_-T2 (r = −0.372, p < 0.01) and rSO_2_-T3 (r = −0.658, p < 0.01). Lactate-T2: Lactate-T2 demonstrates significant negative correlations with both rSO_2_-T2 (r = −0.441, p < 0.01) and rSO_2_-T3 (r = −0.203, p > 0.05). Additionally, there are significant positive correlations with TOS-T2 (r = 0.293, p < 0.05) and OSI-T2 (r = −0.403, p < 0.01), along with significant negative correlations with TOS-T3 (r = −0.417, p < 0.01) and OSI-T3 (r = 0.427, p < 0.01). Lactate-T3: Lactate-T3 shows significant negative correlations with both rSO_2_-T2 (r = −0.468, p < 0.01) and rSO_2_-T3 (r = −0.266, p > 0.05). Moreover, there are significant positive correlations with OSI-T2 (r = −0.324, p < 0.05) and OSI-T3 (r = 0.302, p < 0.05), as well as with Lactate-T2 (r = 0.829, p < 0.01). Htc-T2: Htc-T2 does not exhibit significant correlations with rSO2 values or other oxidative stress biomarkers (all p-values >0.05). Htc-T3: Htc-T3 also does not show significant correlations with rSO_2_ values or oxidative stress biomarkers (all p-values >0.05), except for a positive correlation with Htc-T2 (r = 0.412, p < 0.01). Aortic clamp time: There are significant negative correlations between aortic clamp time and both rSO_2_-T2 (r = −0.349, p < 0.05) and rSO_2_-T3 (r = −0.369, p < 0.01), as well as significant positive correlations with TAS-T2 (r = 0.310, p < 0.05) and TAS-T3 (r = 0.326, p < 0.05). CPB time: CPB time demonstrates significant negative correlations with both rSO_2_-T2 (r = −0.419, p < 0.01) and rSO_2_-T3 (r = −0.501, p < 0.01). Furthermore, there are significant positive correlations with TAS-T2 (r = 0.375, p < 0.01) and TAS-T3 (r = 0.314, p < 0.05), as well as with CPB time itself (r = 0.811, p < 0.01). Duration of surgery: There are significant negative correlations between the duration of surgery and both rSO_2_-T2 (r = −0.487, p < 0.01) and rSO_2_-T3 (r = −0.407, p < 0.01). Postoperative MV duration: Postoperative MV duration exhibits significant negative correlations with both rSO_2_-T2 (r = −0.401, p < 0.01) and rSO_2_-T3 (r = −0.370, p < 0.01). Length of stay in ICU: There are significant negative correlations between the length of stay in the ICU and both rSO_2_-T2 (r = −0.427, p < 0.01) and rSO_2_-T3 (r = −0.189, p > 0.05). Blood transfusion: Blood transfusion demonstrates significant negative correlations with both rSO_2_-T2 (r = −0.512, p < 0.01) and rSO_2_-T3 (r = −0.341, p < 0.05) (Table 4).

#### 3.4.2. Off-pump CABG

The correlation analysis conducted in the off-pump CABG setting revealed several significant associations between intraoperative rSO_2_ values, oxidative stress biomarkers, and other variables. Notably, rSO_2_-T2 showed a significant positive correlation with lactate-T3 (r = 0.185, p < 0.01). Additionally, significant positive correlations were found between rSO_2_-T2 and OSI-T2 (r = 0.894, p < 0.01), as well as rSO_2_-T3 and TAS-T3 (r = 0.271, p < 0.01). No correlation was found between rSO_2_-T2-3 and OS parameters. However, TOS-T3 was strongly positively correlated with the length of stay in the ICU (r = 0.629, p < 0.01) (Table 5).

### 3.5. Independent risk factors for OSIs (T2 and T3) in the LR analysis

When the factors affecting OSIs were analyzed using the multivariate LR backward method (F (2.61) = 8.26, p = 0.001), only lactate-T2 was found to affect OSI-T2 (β = 0.388, p = 0.006; Table 6). Surgery type and rSO_2_-T2 were not statistically significant in the analysis. OSI-T3 was significant with the increase in lactate-T2 (β = 0.317, p = 0.028). Surgery type, rSO_2_-T2, and lactate-T3 were not statistically significant in this analysis.

## 4. Discussion

In this prospective observational study, we compared the relationship between serum OS parameters and rSO_2_ values at different intraoperative time points between on-pump and off-pump CABG procedures. OS values were found to be elevated during both procedures, with a remarkable increase during CPB in the on-pump group. The rSO_2_ values were lower in the on-pump CABG group and showed a negative correlation with OS parameters. Furthermore, elevated intraoperative lactate levels were identified as an independent risk factor for OS.

Our study’s main finding, supported by the literature, reveals an increase in OS parameters during intraoperative time in cardiac surgery, with a greater increase during CPB in the on-pump CABG group [15]. This increase in OS is attributed to tissue damage, inflammatory response, and hemostatic activation, which are considered inevitable tissue injuries related to surgical trauma, regardless of the cardiac surgery type [4,11,15,16]. However, the OS response varies depending on the type of cardiac procedure [5,17,18]. Aortic clamping has been reported to exacerbate OS [19]. Additionally, hypoperfusion, microemboli, and surgical manipulations during CPB can cause myocardial ischemia, resulting in fatty acid oxidation abnormalities and enhanced anaerobic metabolism [3,11]. ROS are also involved in tissue damage after reperfusion [3,19].

In this study, intraoperative oxidant parameters were significantly increased (MDA-T2-3 and TOS-T2-3), while antioxidant parameters were decreased (GPx-T1-2-3 and SOD-T2-3) in the on-pump CABG group compared to the off-pump CABG group. Additionally, OS was positively correlated with aortic cross-clamp time and lactate levels at the T2 and T3 time points in the on-pump CABG group. No such relationship was observed at these time points in the off-pump CABG group. Vukicevic et al. [2] also reported higher OS parameters in the on-pump CABG group, suggesting that OS could be a reliable biomarker for predicting adverse surgical complications. In contrast, another study found that postoperative TOS levels were lower than baseline, and this reduction was significant in the on-pump CABG group. They suggested that this reduction was due to the quick degradation of peroxide (a ROS) [20]. The same study reported that ischemia-modified albumin levels increased more in the on-pump CABG group, suggesting that ischemia may cause an increase in ROS [20]. Gerritsen et al. [17] reported that the level of the lipid peroxidation parameter MDA (an oxidant) was higher in the on-pump CABG group than in the off-pump CABG group during the ischemia and reperfusion stages. Similar to our results, Doğan et al. [15] reported that intraoperative MDA levels in the on-pump CABG group increased significantly compared to the off-pump CABG group relative to the preoperative and postoperative periods. All these findings suggest that CPB, which causes ischemia and reperfusion, contributes significantly to the increase in OS during cardiac surgery.

Conflicting results have been reported regarding the protective effects of SOD on ischemia-reperfusion injury, which is a potent antioxidant against superoxide radicals. While one study has reported an increase in antioxidant parameters after reperfusion [15], others have reported a reduction in those markers [20,21]. Gönenç et al. [18] found that antioxidant GPx levels did not vary according to different time points in either of the groups. That study also found that SOD levels were increased in the on-pump CABG group during ischemia-reperfusion, although they were high in both groups. The authors reported that this increase in SOD activity might be due to the body’s compensatory mechanism to prevent myocardial tissue damage [18]. Vukicevic et al. [2] reported that SOD levels decreased more in the on-pump CABG group compared to baseline. Another study [22] found that the off-pump CABG group exhibited lower TAS levels and perioperative OS parameters. In this study, antioxidant SOD and GPx levels declined at T2 (ischemia) and increased at T3 (reperfusion) time points compared to baseline. However, in the on-pump CABG group, aortic clamp time and the antioxidant parameter TAS were negatively correlated, while oxidant parameters (TOS and OSI) were positively correlated, implying that ischemia-reperfusion during CPB impairs OS. However, there was no clinical difference in the length of ICU stay or MV time due to these variations in OS resulting from different surgical interventions. In contrast, other studies have associated OS with a prolonged postoperative intubation time [8] and increased length of stay in the ICU [1,2].

Cerebral oximetry is used for intervention in cardiac surgery to closely monitor intraoperative cerebral hypoxia or ischemia and prevent adverse clinical outcomes. Numerous published studies have investigated the relationship between rSO_2_ and neurocognitive outcomes in cardiac surgery patients [8–10]. A secondary finding in our study indicates that rSO_2_ levels were lower during CPB in the on-pump CABG group compared to the off-pump CABG group. A similar study [23] reported a reduction in intraoperative rSO_2_ in both groups (more pronounced in the on-pump CABG group), while postoperative rSO_2_ and neurocognitive levels were similar in both groups. The authors of that study attributed this similar postoperative condition to the neuroprotective effects of hypothermia in the on-pump CABG group [23].

Moderate hypothermia, also applied for therapeutic purposes after cardiac arrest, proves beneficial in maintaining cerebral metabolism by concurrently reducing cerebral oxygen consumption and cerebral blood flow in on-pump CABG [7,24]. In this study, moderate hypothermia was used in the on-pump CABG group, resulting in a decrease in rSO_2_ during CPB, consistent with previous studies. However, when examining neurocognitive dysfunction based on cardiac bypass surgery types, Kok et al. [14] reported no difference in cerebral desaturation between on-pump and off-pump cardiac surgery patients.

Our study revealed other significant findings. The rSO_2_-T2 values exhibited a negative correlation with OS parameters. Moreover, lactate levels and aortic clamping time showed a negative correlation in the on-pump group and a positive correlation with antioxidant parameters. These correlations between variables were not observed in the off-pump CABG group. It has been reported that arterial carbon dioxide partial pressure, temperature, pump flow rate, MAP, Hct level, and heart rate affect rSO_2_ during CPB [9]. Patients who are ventilated with supplemental oxygen during CPB and cannot increase rSO_2_ have low cerebral blood flow due to cerebral microemboli [7]. Additionally, the hypothermia used in CPB improves brain metabolism by reducing OS and inflammation [24,25]. Despite these protective mechanisms, we found that low rSO_2_ was inevitable during CPB, and this condition was associated with an increased OS response. However, increased OS during CPB could be both the cause and the outcome of the observed drop in rSO_2_ levels.However, low rSO_2_ levels in cardiac surgery are associated with longer ICU stays, myocardial infarction, stroke, and prolonged postoperative intubation [8]. Although we did not find a correlation between OS levels and clinical outcomes in our study, we did observe a negative correlation between rSO_2_ values during CPB and postoperative MV duration and the length of stay in the ICU only in the on-pump CABG group. Therefore, monitoring cerebral rSO_2_ during on-pump CABG and intervening in disturbances in cerebral perfusion may reduce poor postoperative clinical outcomes.

Lactate, a metabolic intermediate that increases in hypoperfusion, plays a crucial role in the relationship between hypoxia and OS response [11]. Our study revealed that lactate-T2 affected the intraoperative OS response in the regression analysis. Furthermore, in the correlation analysis, lactate-T2-3 levels, aortic clamp time, CPB time, and blood transfusion were negatively correlated with the OS response in the on-pump CABG group. These findings confirm the increased OS response of ischemia-reperfusion during CPB. Therefore, future research may focus on the relationship between OS, rSO_2_, and clinical outcomes such as neurocognitive dysfunction in cardiac surgery.

### 4.1. Strengths and limitations of the study

This study has several strengths. Firstly, unlike some studies that focus solely on rSO_2_ levels based on cardiac surgery type (on-pump and off-pump CABG) [10,23], our study is the first, to our knowledge, to investigate the relationship between intraoperative rSO_2_ and OS). Undoubtedly, further research is warranted to establish more definitive conclusions in this regard. Secondly, a comprehensive analysis of numerous intraoperative variables was conducted for both types of cardiac surgery.

Our study also has several limitations. Firstly, being a single-center study with a relatively modest sample size compared to more extensive published studies, caution is warranted in generalizing our findings. Secondly, postoperative adverse clinical outcomes were limited to MV time and ICU stay length. Future investigations could delve into a broader spectrum of clinical outcomes in this context. Lastly, this study solely measured OS levels. Incorporating measurements of parameters such as inflammatory markers, which are thought to affect OS levels in CPB surgery, could provide additional insights. Future studies should explore the relationships between OS, inflammation, and rSO_2_. Despite these limitations, our results contribute to an important issue in the field, considering the existing literature.

## 5. Conclusion

In summary, OS levels were higher and rSO_2_ levels lower during CPB in the on-pump CABG group compared to the off-pump CABG group. Lower rSO_2_ levels were associated with adverse postoperative clinical outcomes, such as reduced MV time and shorter ICU stay only in the on-pump CABG group. Therefore, we believe monitoring cerebral rSO_2_ during on-pump CABG benefits postoperative clinical outcomes. In addition, the OS response and cerebral oxygenation could be improved by preventing global ischemia in off-pump CABG, improving clinical outcomes. More comprehensive clinical research is needed to better understand the relationship between OS and rSO_2_ in CPB.

## Figures and Tables

**Table 1:** The demographic variables and perioperative characteristics according to on-pump and off-pump CABG groups.

		On-pump CABG (*n* = 48)		Off-pump CABG (*n* = 16)		Total (*n* =64)		p-value
Age (year)	Mean (SD)	61	(10)	59	(11)	60	(10.05)	0.658^b^
Sex n (%)	Male	39	(81.3)	11	(68.8)	50	(78.1)	0.312 a
	Female	9	(18.8)	5	(31.3)	14	(21.9)	
Tobacco use n (%)	Yes	29	(60.4)	10	(62.5)	39	(60.9)	0.882^a^
	No	19	(39.6)	6	(37.5)	25	(39.1)	
Dyslipidemia n (%)	Yes	13	(27.1)	3	(18.8)	16	(25.0)	0.740 a
	No	35	(72.9)	13	(81.3)	48	(75.0)	
Kidney failure n (%)	Yes	2	(4.2)	1	(6.3)	3	(4.7)	0.783^a^
	No	46	(95.8)	15	(93.8)	61	(95.3)	
Hypertension n (%)	Yes	33	(68.8)	12	(75.0)	45	(70.3)	0.758^a^
	No	15	(31.3)	4	(25.0)	19	(29.7)	
Diabetes mellitus n (%)	Yes	25	(52.1)	7	(43.8)	32	(50.0)	0.564
	No	23	(47.9)	9	(56.3)	32	(50.0)	
LVEF (%)	Mean (SD)	52.92	(8)	55.94	(8)	53.67	(8)	0.184^b^
Aortic clamp time (min)	Mean (SD)	59.88	(20)	-	-	59.88	(20)	
CPB duration (min)	Mean (SD)	104.48	(32)	-	-	104.48	(32)	
Total operation time (min)	Mean (SD)	266.77	(73.08)	250.63	(62)	262.73	(70)	0.429^b^
Blood transfusion (U)	Mean (SD)	1.77	(2.1)	0.88	(1.1)	1.55	(2.2)	**0.009** b
ICU MV duration (days)	Mean (SD)	1.25	(0.4)	1.19	(0.2)	1.23	(0.5)	**0.003** b
ICU stay duration (days)	Mean (SD)	3.42	(1.0)	2.50	(1.3)	3.19	(1.2)	0.644^b^

a Fisher’s exact test,

b Independent 2-sample t-test by PND group test p < 0.001.

**Definition of abbreviations:** SD, standard deviation; CPB, Cardiopulmonary bypass; LVEF, Left ventricular ejection fraction; MV, Mechanical ventilation; ICU, intensive care unit.

**Table 2:** Intraoperative oxidative stress parameters according to on-pump and off-pump CABG groups.

	On-pump CABG (*n* = 48)		Off-pump CABG (*n* = 16)			Total (*n* =64)		
	Mean	SD	Mean	SD	p^a^	Mean	SD	p^b^
MDA-T1 (μmol/L)	0.72	(0.10)	0.77	(0.07)	0.064	0.73	(0.10)	**0.011**
MDA-T2 (μmol/L)	1.27	(0.33)	1.07	(0.16)	**0.003**	1.22	(0.31)	
MDA-T3 (μmol/L)	1.36	(0.35)	1.12	(0.20)	**0.001**	1.30	(0.34)	
TOS-T1 (μmol H2O2/L)	8.38	(0.97)	8.99	(0.98)	**0.033**	8.53	(1.00)	**< 0.001**
TOS-T2 (μmol H2O2/L)	13.26	(1.64)	12.12	(1.75)	**0.021**	12.98	(1.73)	
TOS-T3 (μmol H2O2/L)	15.32	(1.70)	13.14	(2.30)	**<0.001**	14.77	(2.08)	
TAS-T1 (mmol trolox equiv./L)	1.65	(0.13)	1.64	(0.13)	0.792	1.65	(0.13)	**0.004**
TAS-T2 (mmol trolox equiv./L)	1.07	(0.22)	1.24	(0.25)	**0.013**	1.11	(0.24)	
TAS-T3 (mmol trolox equiv./L)	1.10	(0.22)	1.31	(0.18)	**0.001**	1.15	(0.23)	
OSI-T1 (AU)	5.12	(0.86)	5.54	(1.00)	0.110	5.23	(0.91)	**0.003**
OSI-T2 (AU)	13.42	(5.12)	10.44	(3.56)	**0.035**	12.68	(4.92)	
OSI-T3 (AU)	15.09	(6.63)	10.33	(2.76)	**0.007**	13.90	(6.24)	
GPX-T1 (U/mL)	135.91	(5.27)	139.36	(3.53)	**0.018**	136.77	(5.09)	0.096
GPX-T2 (U/mL)	117.03	(7.43)	122.29	(4.56)	**0.002**	118.34	(7.17)	
GPX-T3 (U/mL)	118.78	(9.26)	127.00	(7.12)	0.001	120.83	(9.43)	
SOD-T1 (IU/mL)	14.99	(1.34)	15.71	(1.03)	0.054	15.17	(1.30)	0.429
SOD-T2 (IU/mL)	12.26	(1.32)	13.42	(0.70)	**<0.001**	12.55	(1.29)	
SOD-T3 (IU/mL)	12.71	(0.86)	13.85	(0.77)	**<0.001**	13.00	(0.97)	

a Repeated multivariate tests by CABG groups,

b Multivariate tests.

**Definition of abbreviations:** AU, arbitraryunits; Hct, hematocrit; rSO_2_, regional cerebral oxygen saturation; SD, standard deviation; MDA, malondialdehyde; SOD, superoxid edismutase; GPX,glutathion eperoxidase; TAS, total antioxidant status; TOS, total oxidant status; OSI, oxidative stress index; AU, arbitrary units.

**Table 3:** Intraoperative variables according to on-pump and off-pump CABG groups.

	On-pump CABG (*n* = 48)		Off-pump CABG (*n* = 16)			Total (*n* =64)		
	Mean	SD	Mean	SD	p^a^	Mean	SD	p^b^
rSO_2_-T1 (%)	67.75	(5.61)	68.56	(5.06)	0.609	67.95	(5.45)	0.139
rSO_2_-T2 (%)	58.00	(7.35)	62.88	(7.10)	**0.024**	59.22	(7.54)	
rSO_2_-T3 (%)	65.75	(5.59)	67.06	(6.70)	0.442	66.08	(5.86)	
SaO_2_-T1 (mmHg)	97.21	(2.05)	97.30	(1.57)	0.876	97.23	(1.93)	0.403
SaO_2_-T2 (mmHg)	96.87	(3.81)	97.19	(1.48)	0.744	96.95	(3.37)	
SaO_2_-T3 (mmHg)	95.26	(2.17)	96.57	(2.48)	**0.048**	95.59	(2.30)	
PaO_2_-T1 (mmHg)	127.99	(48.77)	136.36	(33.20)	0.526	130.08	(45.28)	0.100
PaO_2_-T2 (mmHg)	165.43	(76.10)	143.61	(21.74)	0.08	159.97	(67.26)	
PaO_2_-T3 (mmHg)	122.94	(50.41)	145.03	(15.38)	**0.009**	128.46	(45.22)	
Lactate-T1 (mmol/L)	1.44	(0.53)	1.30	(0.28)	0.327	1.40	(0.48)	**0.002**
Lactate-T2 (mmol/L)	2.45	(1.39)	1.24	(0.32)	**<0.001**	2.15	(1.32)	
Lactate-T3 (mmol/L)	3.30	(2.14)	1.38	(0.45)	**<0.001**	2.82	(2.04)	
Hct-T1 (%)	38.17	(4.93)	40.13	(3.84)	0.153	38.66	(4.73)	**0.001**
Hct-T2 (%)	26.96	(5.52)	34.69	(4.06)	**<0.001**	28.89	(6.16)	
Hct-T3 (%)	30.69	(3.43)	33.75	(5.74)	0.058	31.45	(4.29)	
Blood glucose-T1 (mg/dL)	126.13	(42.95)	128.56	(37.34)	0.840	126.73	(41.34)	**0.025**
Blood glucose-T2 (mg/dL)	211.10	(54.43)	170.44	(29.05)	**<0.001**	200.94	(52.21)	
Blood glucose-T3 (mg/dL)	233.56	(57.92)	206.75	(56.76)	0.112	226.86	(58.37)	

a Repeated multivariate tests by CABG groups,

b Multivariate tests.

**Definition of abbreviations:** SD, standard deviation; rSO_2_, regional cerebral oxygen saturation; Hct, hematocrit.

**Table 4:** Correlation between intraoperative cerebral oxygen saturation values, oxidative stress biomarkers, and other variables in on-pump CABG.

	rSO_2_-T2	rSO_2_-T3	1	2	3	4	5	6	7	8	9	10	11	12	13	14	15
1. TOS-T2	**−0.312** *	−0.049	1														
2. TOS-T3	**−0.354** *	−0.008	**0.323** *	1													
3. TAS-T2	0.216	0.124	**−0.557** **	**−0.411** **	1												
4. TAS-T3	**0.405** **	0.019	**−0.372** **	**−0.658** **	**0.514** **	1											
5. OSI-T2	−0.259	−0.071	**0.691** **	**0.498** **	**−0.931** **	**−0.602** **	1										
6. OSI-T3	**−0.377** **	−0.061	**0.366** *	**0.755** **	**−0.560** **	**−0.907** **	**0.693** **	1									
7. Lactate-T2	**−0.441** **	−0.203	**0.293** *	0.275	**−0.403** **	**−0.417** **	**0.427** **	**0.404** **	1								
8. Lactate-T3	**−0.468** **	−0.266	0.237	0.187	−0.224	**−0.324** *	0.251	**0.302** *	**0.829** **	1							
9. Htc-T2	0.110	−0.016	−0.012	−0.129	−0.132	0.065	0.059	−0.143	0.165	0.087	1						
10. Htc-T3	−0.064	−0.012	0.114	0.064	−0.148	0.004	0.161	0.013	0.138	0.030	**0.412** **	1					
11. Aortic clamp time	**−0.349** *	**−0.369** **	**0.310** *	**0.326** *	**−0.421** **	**−0.456** **	**0.440** **	**0.456** **	**0.583** **	**0.498** **	−0.105	0.071	1				
12. CPB time	**−0.419** **	**−0.501** **	0.254	0.200	**−0.351** *	**−0.378** **	**0.375** **	**0.314** *	**0.648** **	**0.511** **	0.046	0.159	**0.811** **	1			
13. Duration of surgery	**−0.487** **	**−0.407** **	0.135	0.149	−0.164	−0.200	0.164	0.146	**0.722** **	**0.662** **	0.215	**0.338** *	**0.492** **	**0.664** **	1		
14. Postoperative MV duration	**−0.401** **	**−0.370** **	0.209	0.147	−0.188	−0.162	0.213	0.166	**0.628** **	**0.522** **	0.132	**0.509** **	**0.355** *	**0.565** **	**0.757** **	1	
15. Length of stay in ICU	**−0.427** **	−0.189	0.157	0.221	−0.061	−0.211	0.084	0.190	**0.527** **	**0.459** **	−0.247	0.249	**0.519** **	**0.517** **	**0.539** **	**0.507** **	1
16. Blood transfusion	**−0.512** **	**−0.341** *	0.157	0.045	−0.107	−0.259	0.106	0.163	**0.522** **	**0.679** **	−0.001	0.028	**0.492** **	**0.556** **	**0.526** **	**0.384** **	**0.408** **

* Correlation is significant at the 0.05 level (2-tailed).

** Correlation is significant at the 0.01 level (2-tailed).

**Definition of abbreviations:** CPB, Cardiopulmonary bypass; MV, Mechanical ventilation; rSO_2_, regional cerebral oxygen saturation; Hct, hematocrit; TAS, total antioxidant status; TOS, total oxidant status; OSI oxidative stress index

**Table 5:** Correlation between intraoperative cerebral oxygen saturation values, oxidative stress biomarkers and other variables in off-pump CABG.

	rSO_2_-T2	rSO_2_-T3	1	2	3	4	5	6	7	8	9	10	11	12	13	14
1. TOS-T2	0.047	0.182	1													
2. TOS-T3	−0.247	0.271	0.178	1												
3. TAS-T2	−0.054	0.183	**−0.814** **	0.006	1											
4. TAS-T3	−0.048	0.095	−0.445	−0.373	0.472	1										
5. OSI-T2	−0.046	−0.071	**0.894** **	0.062	**−0.963** **	−0.463	1									
6. OSI-T3	−0.119	0.083	0.362	**0.826** **	−0.219	**−0.809** **	0.270	1								
7. Lactate-T2	−0.428	0.132	−0.074	0.261	−0.050	−0.037	0.057	0.113	1							
8. Lactate-T3	−0.405	0.185	0.148	0.399	−0.239	−0.083	0.250	0.216	**0.910** **	1						
9. Htc-T2	−0.476	−0.200	0.135	0.261	0.044	−0.167	0.115	0.341	0.059	0.058	1					
10. Htc-T3	**−0.517** *	−0.013	0.061	0.287	0.134	−0.093	0.041	0.308	0.230	0.176	**0.925** **	1				
11. Duration of surgery	−0.387	−0.024	−0.413	−0.039	0.426	**0.627** **	−0.354	−0.333	0.224	0.094	0.122	0.285	1			
12. Postoperative MV duration	−0.178	0.069	−0.127	0.447	0.342	−0.240	−0.246	0.495	0.097	−0.065	0.364	0.367	0.366	1		
13. Length of stay in ICU	−0.371	−0.039	0.056	**0.629** **	0.021	−0.033	−0.003	0.441	0.211	0.252	0.143	0.147	0.490	**0.654** **	1	
14. Blood transfusion	0.029	0.069	0.126	0.115	0.062	−0.303	−0.059	0.285	0.380	0.312	0.248	0.334	−0.258	0.070	−0.119	1

* Correlation is significant at the 0.05 level (2-tailed).

** Correlation is significant at the 0.01 level (2-tailed).

**Definition of abbreviations:** CPB,Cardiopulmonary bypass; MV, Mechanical ventilation; rSO_2_, regional cerebral oxygen saturation; Hct, hematocrit; TAS, total antioxidant status; TOS, total oxidant status; OSI oxidative stress index.

**Table 6:** Analysis of factors affecting oxidative stress indexes (T2, T3) by linear regression

aOSI-T2: Adj.R^2^=0.181, F (3.60)=5.63, p=0.002	Unstandardized coefficients		Standardized coefficients	t	p	95.0% confidence interval for B	
	B	Std. error	Beta			Lower bound	Upper bound
(Constant)	12.510	5.602		2.233	**0.029**	1.306	23.715
Lactat-T2	1.442	0.507	0.388	2.843	**0.006**	0.427	2.456
b **OSI-T3: Adj.R** **^2^** **=0.249, F (5.58)=5.17, p=0.001**							
(Constant)	26.353	7.561		3.486	**0.001**	11.218	41.487
Lactat-T2	2.306	1.025	0.486	2.250	**0.028**	0.254	4.358

Definition of abbreviations: OSI, oxidative stress index

**Independent variables:**

a CABG bypass type, rSO_2_-T2, lactat-T2;

b bypass type, rSO_2_-T2, lactat-T2, lactat-T3.
